# Inception Convolution and Feature Fusion for Person Search

**DOI:** 10.3390/s23041984

**Published:** 2023-02-10

**Authors:** Huan Ouyang, Jiexian Zeng, Lu Leng

**Affiliations:** 1 School of Software, Nanchang Hangkong University, Nanchang 330063, China; 2 Key Laboratory of Jiangxi Province for Image Processing and Pattern Recognition, Nanchang Hangkong University, Nanchang 330063, China; 3 Science and Technology College, Nanchang Hangkong University, Gongqingcheng 332020, China

**Keywords:** person search, Faster R-CNN, inception convolution, feature fusion, region proposal network (RPN), double-head, efficient learning

## Abstract

With the rapid advancement of deep learning theory and hardware device computing capacity, computer vision tasks, such as object detection and instance segmentation, have entered a revolutionary phase in recent years. As a result, extremely challenging integrated tasks, such as person search, might develop quickly. The majority of efficient network frameworks, such as Seq-Net, are based on Faster R-CNN. However, because of the parallel structure of Faster R-CNN, the performance of re-ID can be significantly impacted by the single-layer, low resolution, and occasionally overlooked check feature diagrams retrieved during pedestrian detection. To address these issues, this paper proposed a person search methodology based on an inception convolution and feature fusion module (IC-FFM) using Seq-Net (Sequential End-to-end Network) as the benchmark. First, we replaced the general convolution in ResNet-50 with the new inception convolution module (ICM), allowing the convolution operation to effectively and dynamically distribute various channels. Then, to improve the accuracy of information extraction, the feature fusion module (FFM) was created to combine multi-level information using various levels of convolution. Finally, Bounding Box regression was created using convolution and the double-head module (DHM), which considerably enhanced the accuracy of pedestrian retrieval by combining global and fine-grained information. Experiments on CHUK-SYSU and PRW datasets showed that our method has higher accuracy than Seq-Net. In addition, our method is simpler and can be easily integrated into existing two-stage frameworks.

## 1. Introduction

Person search [1,2] is a major area of research in the field of computer vision. It can take a range of untreated images from surveillance and video and then locate and identify people in the images. Due to the difficulty of pedestrian detection and pedestrian re-identification (re-ID) involved in the process of person search [3,4], the existing methods of pedestrian retrieval can generally be classified into two categories: the two-step method and the one-step method. In the two-step method [5,6], pedestrian retrieval is split into two separate areas of person search [7,8] and pedestrian re-identification (re-ID) for research, but the two-step method has many problems in practical use; for example, it takes too long and consumes resources [9]. In contrast, the one-step method [10,11] combines person search and pedestrian re-identification [12,13] modeling, and the research in a single framework enables the two components to communicate information [14,15], improve one another, and produce superior results.

In recent years, with the rapid development of information technology and network data, image acquisition has improved in quality. People pay more and more attention to social security issues. Security measures are becoming more and more effective. In addition, video surveillance areas are becoming more widespread. Every moment generates a large amount of surveillance video. The reality of accurate person search is a huge challenge. Social security and public security need to correctly identify pedestrians in complex scenes in order to solve crimes. Re-ID methods are mainly used in traditional environments with ample light and people in bright clothing, and the re-ID model is also primarily based on the clothes of people. In real business scenarios, we find that, in winter, the performance of the re-ID system suddenly drops significantly; thus, improving the accuracy of person search in real scenarios is a topic well worth studying.

The problems to be solved by person search include pedestrian detection and pedestrian re-identification, which not only need to consider the improvement of accuracy, but also the real-time requirements of some application scenarios and the limitations of the equipment. As a result, researchers aim to better balance network models’ speed and precision. After years of development, the Faster R-CNN [16] based target detection algorithm can now achieve high performance while also slowing down detection. Some algorithms for pedestrian retrieval [17] with deeply integrated networks [18] have achieved reasonable performance. For the one-step approach, they designed a multi-tasking framework [19] based on Faster R-CNN, establishing a regional proposal network (RPN) to generate region proposal [20] and then input it into subsequent parallel detection and re-ID branches. However, these features extracted from the network inevitably introduce location deviations, and some even extract poor features. These factors considerably lower the efficiency of fine-grained re-ID while having minimal impact on the work of coarse-grained classification.

**Figure 1:**
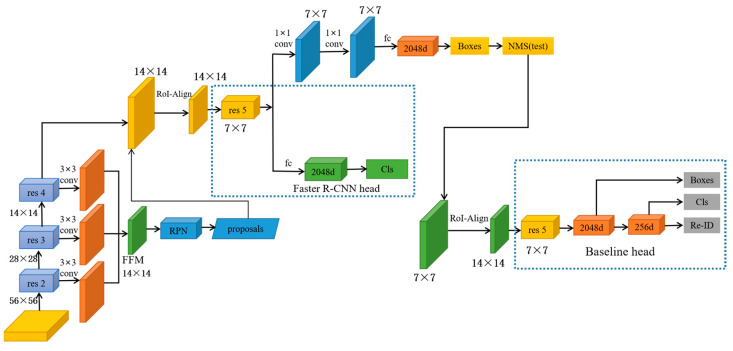
The inception convolution and feature fusion frame diagram.

Motivated by the above observations, the performance of later re-ID can be negatively impacted by subpar and erroneous features acquired by Faster R-CNN. Therefore, to extract high-quality features, we propose a person search approach based on an inception convolution and feature fusion module (IC-FFM), which is presented in Figure 1. The ResNet-50 network structure consists of 49 convolutional layers and one fully connected layer, and the specific network structure can be divided into seven parts. The first part does not contain residual blocks, which are mainly used for the calculation of convolution, regularization, activation function, and maximum pooling of the input, and the second, third, fourth, and fifth parts of the structure contain residual blocks that do not change the size of the residual blocks but are only used to change the dimensionality of the residual blocks. ResNet-50 is a residual network with lower complexity, more stable performance [18], and faster convergence compared to VGG 16, and it is suitable for many projects with more accurate results in image classification, target detection, and natural language processing. First, we replaced the general convolution in ResNet-50 with inception convolution in Seq-Net, dynamically enhancing the receptive field of feature diagrams [20,21] without increasing computation or degrading feature diagram resolution. Then, the res 2, res 3, and res 5 obtained through inception convolution are convolved 3 × 3, respectively, followed by feature fusion. Without increasing the thickness of the candidate box extract layer, we improved the reliability of target identification and positioning with image detail information in the lower feature layer combined with abstract feature information [22] in the higher feature layer. Finally, to further enhance the effectiveness of detector classification and regression, Bounding Box regression by convolution is performed using the double-head module (DHM).

Our proposed method (IC-FFM) has some improvements over several existing mainstream two-stage pedestrian retrieval models. On the CHUK-SYSU dataset, our mAP value is 96.2%, and on the PRW dataset, our mAP value is 48.9%. On the CHUK-SYSU dataset, our method has a 1% improvement compared to PS-ARM, a 1.4% improvement compared to Seq-Net [23], and a 3.2% improvement compared to OIM [24]; on the PRW dataset, our method has a 0.3% improvement compared to PS-ARM [25], a 1.3% improvement compared to Seq-Net, and a 6% improvement compared to OIM.

To summarize, our proposed work makes the following contributions:

- We devised the inception convolution module (ICM) that has the ability to sequentially mine all conceivable salient characteristics and merge these discriminative salience features with the global feature to obtain more bottom information of different pedestrian features.
- We devised a feature fusion module (FFM). In order to improve the network representation capability and the acquisition of underlying information, this module can combine parallel feature layers of various resolutions. It can also compactly extract global and local appearance information, stack structural information, and change spatial relationships to create new features, and it can use that information as input for the following stage.
- We proposed a double-head module (DHM) to further enhance the effectiveness of detector classification and regression. The DHM is a multi-aspect feature extraction mechanism that can use the operation of dynamic convolution to capture long-distance features to enable a stronger representation learning ability to extract richer pedestrian features.
- In the Faster R-CNN branch, we developed a high-performance, effective information fusion method to extract rich contextual and fine-grained information. Extensive experiments and visual comparisons on CUHK-SYSU [26] and PRW [27] demonstrated that our method IC-FFM is efficient and reasonable and significantly outperforms existing state-of-the-art methods.

## 2. Related Works

Person search technology can be divided into five steps: data gathering, bounding box construction, training data annotation, model training, and person search. Numerous deep learning-based approaches to solving pedestrian re-identification tasks [23] have been created recently as a result of ongoing advancements in computing power. In this section, we introduce a few examples of works related to ours.

### 2.1. Pedestrian Detection

Pedestrian detection is a typical target detection that aims to determine whether a target of interest appears in an image and pinpoint it by computer vision techniques. In recent years, due to the successful application [28] of deep learning in the field of computer vision, algorithms based on convolutional neural networks have also been gradually applied to pedestrian detection. Some pedestrian detection works use R-CNN [10] as an architecture to perform classification and rely on ICF [29] to generate candidate frames. In particular, the one-stage target detection algorithms represented by YOLO [12] and SSD [30] and the two-stage algorithms [16] represented by Faster R-CNN occupy the dominant position in the target detection field. Pedestrian detection is a very critical step in person search, and low-quality candidate frames and features can greatly reduce the accuracy of later pedestrian detection and have an impact on the overall person search framework. Faster R-CNN solves the problem of slow generation of candidate frames by adding RPN (Region Proposal Network) to the network, which first generates and screens the candidate frames and then performs optimization of candidate frame positions and category prediction. Though Faster R-CNN can perform classification well, its features lose translation undistorted when operated with Rol Pooling, which can lead to some reduction in detection accuracy. In order to solve these problems, in this paper, we design a model framework for the pedestrian detection part based on Faster R-CNN to achieve better performance by modifying some components.

### 2.2. Pedestrian Re-Identification

Pedestrian re-identification [22] is a technique that uses computer vision techniques to discriminate the presence of a specific pedestrian in an image or video frame sequence and has been widely studied since Gheissari et al. In recent years, some traditional methods, such as Liao et al., proposed to use a combination of local maximum occurrence feature extraction (LOMO) [31] and metric-based cross-quadratic discriminant analysis (XQDA) [32] to extract pedestrian features and obtain relatively accurate results. Chen et al. proposed a unified framework that uses similarity functions [33] and polynomial feature mapping to handle the representation and matching of different subregions. Thanks to the powerful feature representation capability of convolutional neural networks (CNN) (ResNet, DesNet), Li et al. combined twin networks and CNN networks [11] for the first time to solve the non-alignment problem in pedestrian re-identification. After realizing the bottleneck of global-based features, researchers started to turn their attention to local features. Wang et al. sliced ResNet-50 into multiple branches and learned multi-grain features of pedestrians from them, which is a simple and effective method. Sun et al. proposed (VPM) [34] to solve the alignment problem by extracting region-level features and comparing features at the same location in two images. Chen et al. modeled and counted the higher-order information [35] in the attention mechanism to capture the nuances between pedestrians. Zhou et al. used the attention mechanism to obtain pedestrian masks and normalized the masks [36] obtained from different depth network layers to make the network more accurate in capturing pedestrian foreground information.

Pedestrian re-identification is the last and most significant step in the person search framework. In simple terms, in the person search framework, the purpose of pedestrian re-identification is to perform the process of feature extraction twice and then feature matching, and the final matching accuracy varies for different matching methods. The first matching method is to treat different pedestrians as different classes and to obtain a classification problem and deep network for feature extraction, followed by a softmax layer classification; the second way is to use triplet loss to increase the feature distance between classes and reduce the feature distance within classes; the third method is to conduct local matching. Each person is divided into multiple parts (can be understood as head, body, legs), each part and classification is analyzed, and finally, they are combined together to determine the class of the person. However, the parallel structure of the Faster R-CNN extracts features from low-quality proposals generated by the regional proposal network, rather than the detected high-quality bounding boxes. Person search is a fine-grained task, and these poor quality features significantly degrade the performance of re-ID. To address the above issues, this paper proposes a new feature extraction module to obtain higher quality bounding boxes for better results in the final pedestrian re-identification task.

### 2.3. Person Search

Person search aims to detect and identify the identity to be retrieved directly from all the original surveillance video frames and is an extension of pedestrian re-identification techniques. In the two-step method, pedestrians are first detected and then fed into a re-ID model for recognition, i.e., detection and re-ID models [37] are independent of each other, while in the one-step method, pedestrian detection and re-ID are combined and implemented together. Xu et al. first proposed the concept of pedestrian retrieval [38] in 2014 to implement a model that combines pedestrian detection and pedestrian recognition; however, the results on large-scale datasets are not satisfactory.

The one-step method based on Faster R-CNN is also popular, Xiao et al. proposed a unified network [39] using an online instance matching loss function (OIM) [40] to optimize detection and recognition. The network can optimize the expected log-likelihood by calculating the probability of labeled and unlabeled identities’ online instance matching loss, which can take into account the unlabeled identities [41] in the dataset and achieve better results. Liu et al. constructed a natural detection network (NSN) [42] that can preserve spatial information from spatio-temporal sequences based on a convolutional long-short network [43] (Conv-LSTM), which can integrate the pedestrian features to be queried into the network memory and target the location of the target task by recursively narrowing the region to be retrieved. This is a novel approach that eliminates the step of pedestrian detection by combining contextual information, and it can eliminate the error accumulation effect of pedestrian detection. By introducing a central loss function [44] (Center Loss), Xiao et al. proposed an independent aggregation network with three stages using different loss functions [45] to fine-tune the network to achieve optimal results. Chen et al. optimized the feature differentiation method [35] by transforming features to polar coordinates so that the background has as little effect on pedestrian features as possible and achieved good results by optimizing feature distances [41] in different spaces. Han et al. designed an ROI [46] (region of interest) transform layer, which effectively implements the transformation of the detection frame [42] from the original image to achieve end-to-end training. Chen et al. proposed Hierarchical Online Instance Matching (HOIM) [40] loss, which pairs detection and re-ID to guide feature learning in their network. Dong et al. designed a two-way interaction network (BINet) [47] to remove the returned contextual information. To reconcile the conflicting goals of these two subtasks, Chen et al. proposed a new method called norm-aware embedding (NAE) [46] to embed people into norms and angles for detection and re-identification, respectively. Li et al. proposed a Sequential End-to-End Network (Seq-Net) [48] to extract superior features.

In fact, the person search task still has many pressing problems, such as pedestrian detection frames of different sizes or inaccurate positioning, speckled and complicated background information in the pedestrian frames, and obstructed pedestrians. This network introduces a large detection error when performing feature extraction, which attracts too much unnecessary background information and affects the network’s ability to extract more refined pedestrian features. At the same time, the size of pedestrian frames in real scenes varies greatly, which can lead the network to extract impure pedestrian features, and the existing pedestrian feature extraction methods may generate results at different semantic levels, which seriously affects pedestrian re-identification accuracy. All these problems can significantly impede the extraction of pedestrian features from discriminative rows, which impacts the accuracy of pedestrian re-recognition results. To address the above issues, this paper improved a novel network structure and also proposes a new retrieval method that yields more useful results in pedestrian retrieval tasks.

## 3. Proposed Method

We propose a pedestrian retrieval algorithm based on an inception convolution and feature fusion module (IC-FFM). The mechanism consists of three main modules: the new cavity convolution variant module (ICM), the feature fusion module (FFM), and the double-headed mechanism module (DHM), and the framework is shown in Figure 1. In this paper, the proposed network first performs three 1 × 1 convolution operations on the feature maps. These operations obtain 56 × 56 res 2, 28 × 28 res 3, and 14 × 14 res 4 feature maps and then fuse them to obtain the 14 × 14 FFM module. The RoIAlign module is used twice. This is the first time a pedestrian global feature has been extracted whose size is 14 × 14. The second time is to output features to the pedestrian key node detection branch with a size of 14 × 14 and, finally, to the pedestrian re-identification branch of the baseline. ICM is a new inception convolution to solve the problem of insufficient feature map perceptual field in the backbone network. FFM is used to manipulate the features in each layer of the backbone network passing through ICM, combining the image detail information in the lower layers and the feature information in the higher layers to improve the reliable new target identification and localization. The double-head module (DHM) is used to perform classification combined with regression at the same time to further improve the performance of the detector for classification and regression.

### 3.1. Inception Convolution Module

The person search algorithm is a combination of pedestrian detection and pedestrian re-identification, aiming to obtain both pedestrian location and pedestrian identity by one forward prediction. However, in dense scenes, since pedestrians are non-rigid objects, their poses are changeable, and there are problems of small scale and occlusion, the detector needs to extract stronger pedestrian features to reduce the missed detection rate and improve the accuracy. Moreover, the pedestrian features extracted by ordinary convolution are limited due to their own characteristics. The process of feeding images into the convolutional network performs successive operations on them, including convolution, pooling, and down-sampling, to integrate multi-scale contextual information. This method can be used to learn more about local features, but it ignores the contribution of other regions to the current region, which leads to some loss of resolution and leads to some details that cannot be reconstructed, which makes person search difficult.

The receptive field is a very significant concept in convolutional neural networks. In fact, only a few pixels around the center of the receptive field can contribute effectively to the output neuron. Therefore, setting different receptive fields [49] for different tasks [33] can effectively improve the accuracy of object detection. It is typically implemented using dilated convolution, but it is very significant how the hole rate of the dilated convolution is assigned.

**Figure 2:**
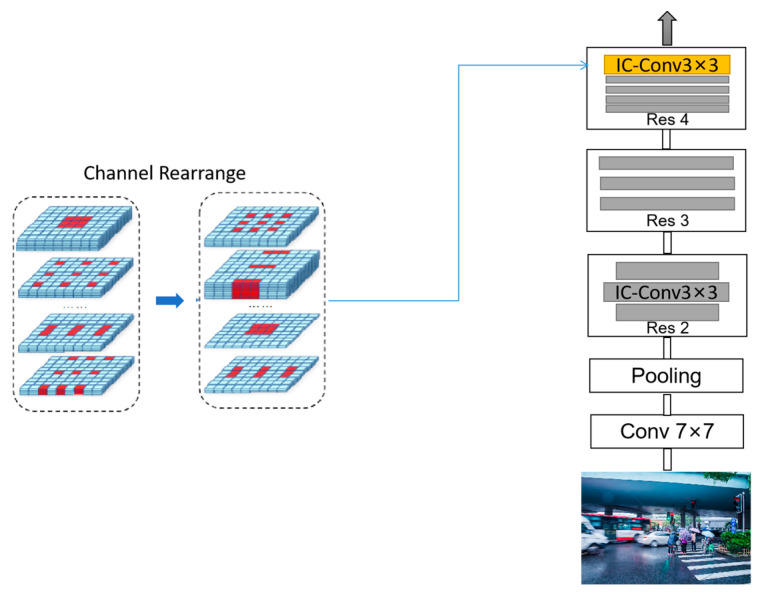
The inception convolution module.

Inspired by the above work, this paper adopted the inception convolution module (ICM) instead of general convolution in ResNet-50. This convolutional approach with a very flexible search space can effectively improve the ability to fit the effective perceptual field to different data sets. It also allows the convolution operation to dynamically and efficiently assign the perceptual field to different channels, which can expand the perceptual domain of the feature map without increasing the computational effort and losing the resolution of the feature map. This can result in obtaining rich contextual information and enhancing the performance of pedestrian retrieval and the detection of obscured pedestrians. First, we changed the kernel size for $(2d_{max}+1)\times \left(2d_{max}+1\right)$ for the convolutional layer in ResNet-50 to a kernel size of 3 × 3. In our experiments, we set $d_{max}=2$, and the kernel size of the super net was 5 × 5. Second, we chose the optimal expansion pattern with the most optimal representation capability, which resulted in the smallest representation error *E* (see Equations (2) and (3)). For one channel, the expansion using *E* = 3 was chosen in this paper. Finally, we rearranged the filters so that the filters with the same dilation pattern were aligned together, which produced our ICM. The structure of the ICM is shown in Figure 2.

Multiple convolutional layers are stacked in a conventional CNN to improve the receptive domain. In order to aggregate various receptive domains, the first network incorporates filters of various sizes that function at the same level. We considered a complete expansion space to alter the issue of low normal convolutional resonance fields. This is how the form is expressed:

(1) $$d=\left\{(d_{x}^{i},d_{y}^{i}|d_{x}^{i}\in \left\{1,2,\ldots d_{max}\right\}\right\},d_{y}^{i}\in \left\{1,2,\ldots d_{max}\right\},i\in \left\{1,2,\ldots ,C^{out}\right\}$$

where $C^{out}$ stands for the number of output channels, $d_{x}^{i}$ and $d_{y}^{i}$ are the filter’s *x* axis and *y* axis dilation values for the *i* th output port, respectively, ranging from 1 to $d_{max}$. In a single initial convolution, there are a maximum number of expansion modes $(d_{max}{}^{2C_{out}}$). In this research, we show that our ICM can automatically select the best *d* to quickly learn the best receiving domain for various jobs.

In order to cover all potential extension patterns for a given network architecture, we first construct a super-network while maintaining the network’s original architecture. Officially, we substitute a convolutional kernel of size $2kd_{max}+1$ for a supergrid convolutional layer with kernel size 2*k* + 1. The dimension corresponds to the most diverse and extensive potential extension mechanisms. It is worth noting that the superior network is pretrained for each activity.

For each convolutional layer with weight *W*, we define the weights of the $W^{i}$ of convolutional filter and denote the set of $\left\{(d_{x}^{i},d_{y}^{i})|x_{i=1}^{C_{out}}\right\}$; $d_{x}^{i}$ and $d_{y}^{i}$ represent the sampling locations of channel *i*-th. We stack $W^{i}{}_{d_{x}^{i}d_{y}^{i}}$ along the output channel dimensions and generate $d_{x}^{i}$ and $d_{y}^{i}$, which are controls, and $C_{out}$ is the number of input and output channels. Note that the dimensions of *W* and $W_{d}$ are the same, and $W^{i}$ and $W^{i}{}_{d_{x}^{i}d_{y}^{i}}$ are also the same, where $C_{in}$ and $C_{out}$ are the number of input and output channels. We represent the expansion mode selection task as an optimization problem where the *L_1_* error between the output expectation of the pre-trained weights *W* and the output expectation of the sampled expansion weights $W_{d}$ is minimized. ICM can be obtained as follows:

(2)  dmin||E|W∗X|−E[Wd∗X]||1s.t. dxi∈{1,…,dmax},dyi∈{1,…,dmax}

where *X* ∈ $R^{B\times C_{in}\times H\times W}$ is the input for this convolutional layer, which has the following parameters: batch size *B*, number of input channels $C_{in}$, height *H*, and width *W*. The convolution operator is represented by ∗, and the expectation operator is represented by *E*. In the following formula, formula (2) can be further expressed as:

(3) ||E[W∗X]−E[Wd∗X]||1=||W∗E[X]−Wd∗E[X]||1=||(W−Wd)∗E[X]||1= dmin∑i=1Coutα||(Wi−Wdxi,dyi)∗1||1

The “1” is an integral matrix with the same dimension as *X*, and *α* is a constant scalar that can be omitted in optimization. The optimal d of each convolution layer can be easily solved by the expansion mode $\left(d_{x}^{i},d_{y}^{i}\right)$ of each filter $W^{i}$.

In the ordinary initial convolution ${\left(d_{max}\right)}^{2}$, operations are computed sequentially, while our ICM module can compute ${\left(d_{max}\right)}^{2}$ operations in parallel. In addition, the total cost of ordinary convolution is $C_{in}\times C_{out}\times {\left(2k+1\right)}^{2}\times {\left(d_{max}\right)}^{2}$, but the total cost of our ICM is $C_{in}\times C_{out}\times {\left(2kd_{max}+1\right)}^{2}$. For most CNNs, where *k* is usually 1, when *dmax* equals 4, $C_{in}\times C_{out}\times {\left(2kd_{max}+1\right)}^{2}$ is only 56% of $C_{in}\times C_{out}\times {\left(2k+1\right)}^{2}\times d_{max}{}^{2}$. Therefore, our ICM is more effective than the ordinary convolution. The experimental results show that the ICM proposed in this paper is effective in obtaining rich contextual information. Moreover, it can be used in different neural network frameworks. Most importantly, it can improve the accuracy of the later re-ID.

### 3.2. Feature Fusion Module

Different feature layers have different information after feature fusion, and integrating feature layers with different resolutions improves network representation and enhances the acquisition of underlying information. The fusion and analysis of various layers of features facilitate semantic segmentation and target detection. The extracted features have limitations because the standard convolutional kernel is spatially invariant and localized, and the learned parameters are static. Several studies have proposed novel feature fusion methods, such as attention mechanisms, deeper and wider networks, dynamic convolution, and multi-scale feature fusion. Common fusion operations are performed at the common pixel level, such as addition or concatenation, but these methods have limited performance improvement and lack semantic information.

**Figure 3:**
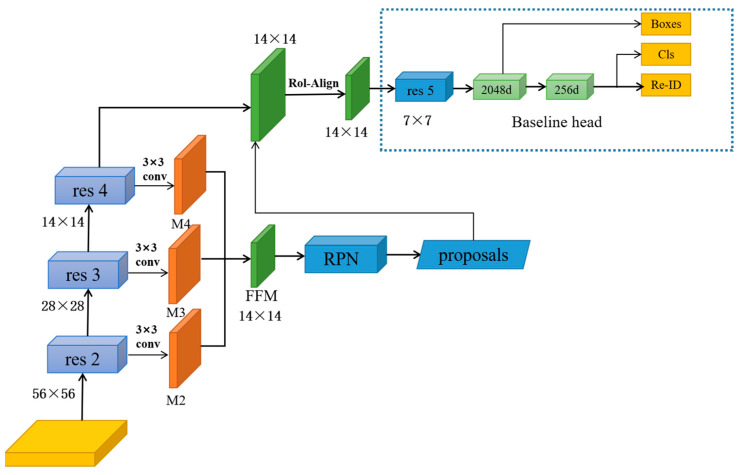
The feature fusion module.

The RPN network mainly learns two types of information: (i) category information, according to the candidate region feature information to calculate the candidate region category information and to determine whether it contains the target, and if it does, it is the foreground class, otherwise, it is the background class; (ii) location information, i.e., the target location border is regressed from the candidate region border. In fact, RPN does not directly train to learn the location information itself, but it learns the offset of the candidate region borders relative to the target location borders. Traditional RPN uses the deep feature layer as the potential frame extraction layer. This results in more loss of imagery complete details in the deep feature layer and also more loss of objective feature information after multiple wavelet decomposition, which results in targeting errors. To merge characteristics from various levels of varying sizes and maintain features in the final approximation, we devised an FFM, as illustrated in Figure 3. This is motivated by the long-term dependency on the process of merging several layers of features.

We utilize the additional relevant details in the res2 and res3 feature layers for subsequent image retrieval using 3 × 3 convolution kernels. All of that is performed in order to create two additional feature layers, M2 and M3. The feature layer res4 is then convolved using 3 × 3 convolution kernels to produce feature layer M4, and M4 is then merged together with feature layers M2 and M3 to produce a new feature layer FFB, which is utilized as the ultimate prospective frame extraction layer and then supplied to the RPN module. Because FFB contains the same thickness as M4, this method can enhance the trustworthiness of object recognition and localization by incorporating image complete details from the relatively low feature layer with abstract feature information from the higher feature map without varying the thickness of the candidate frame separation layer. In this essay, the feature layer M4 is merged with the feature layers M2 and M3 to produce the feature layer FFM, in which the obtained features of three feature layers with the same length and width at the correct locations are incorporated to create a brand-new individual feature with the same number of feature values. Following the determination of the applicant frame extraction layer, the applicant frames are retrieved by the RPN network and supplied to the classification model and boundary reversal network. This enables target detection function training and boundary recurrence function training, respectively, in a similar manner to conventional RPN network training.

Res2-res4 represents different levels of feature inputs, where the bottom feature map is larger and contains more feature information, while the higher level feature maps are smaller in size but have larger objects. Through research experiments, it is found that, as the objects become larger, the feature maps fused by FFM can obtain more information and have superior performance in recognizing pedestrians.

### 3.3. Double-Head Module

In dense scenes, since pedestrians are non-rigid objects with variable poses and small scale and occlusion problems, the detector needs to extract stronger pedestrian features to reduce the miss detection rate and improve accuracy. When utilizing Faster R-CNN in a ResNet-50 backbone to retrieve a feature map, the produced feature maps are essentially single-layered with generally tiny resolutions, and feature recovery performance is low for multi-scale and low targets. We discovered that two frequently utilized head architectures (convolutional head and fully connected head) exhibit opposing inclinations for classification and localization objectives in object detection throughout our studies. Regarding them, fc-head is better suited for classification, whereas conv-head is better suited for localization. Furthermore, we evaluated the output feature maps of both heads and discovered that the fc-head had superior spatial selectivity than the conv-head. Thus, the fc-head has more ability to distinguish a complete object from a part of an object, but it cannot regress the whole object robustly. The improvement of the sparse detection head can further improve the accuracy of classification and regression. Inspired by this, we considered whether using 2 heads for classification and regression at the same time will produce better results.

**Figure 4:**
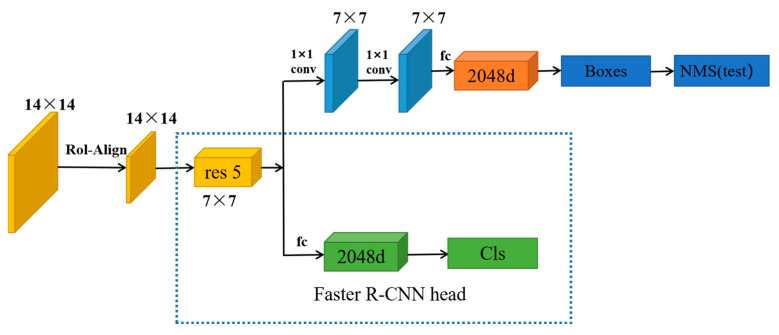
The double-head module.

In Seq-Net, we found that it picks up a 2048-dimensional full connection after the res5 feature map and then performs classification and regression before. However, in our experiments, we found that RPN generates overlapping candidate frames in the generated proposals, which is very detrimental to the extraction of fuzzy targets. Introducing twice the original ROI of Fast R-CNN leads to a loss of accuracy. Therefore, we cannot focus only on critical information when detecting output. Instead, we need to search globally. However, this consumes training time, and it is difficult to find the critical part. Using two detection heads for Bounding Box regression would be better than using full connectivity. This is because full connectivity is more sensitive to spatial location and is better for extracting information. Therefore, in this paper, we used a double-headed mechanism module (DHM) to perform classification and regression simultaneously, as shown in Figure 4.

We constructed regions of interest using the FPN backbone and captured object characteristics at several levels using RoIAlign. Each proposal contains a feature map of size 256 × 7 × 7, which is translated into two feature vectors (of size 1024, respectively) for categorization and bounding box regression with fc-head and convex head, respectively. We use the double-head algorithm to perform both classification and regression prediction by means of two logical-join operations. One branch uses the same two full-join as the CrowdDet algorithm to complete the classification task; the other branch applies full convolution to complete the regression task. First, we Rol-Align the original imagery to obtain the extracted features into res5, then we use two 7 × 7 convolutions of the behind res5 feature map for the bounding box regression, two 2048-dimensional complete merges for the objective categorization, and lastly access the re-ID head to complete the re-identification operation.

We use the double-head mechanism module (DHM) method, focusing on the fully connected head for classification and the convolutional head for bounding box regression to improve the accuracy of later re-ID. Extensive experiments validate the effectiveness of the method.

## 4. Experiments and Results

### 4.1. Experimental Equipment and Datasets

We used the Ubuntu 16.04 System, and the training hardware was two 2080Ti graphics cards with 11GB of video memory; then, the test results were obtained on one 2080Ti. We used the CUHK-SYSU dataset and PRW dataset in the experiment. The experiments were performed using the backbone network of ResNet-50 pre-trained on ImageNet, using SGD stochastic gradient descent for 19 epochs (15 epochs for PRW), with a momentum value of 0.9, a decay factor of 0.9, an initial learning rate of 0.006 on both PRW and CUHK-SYSU datasets, and a decay of 10 on the 16th epoch, with SGD momentum decayed to 0.9, the weights decayed to $5\times 10^{-4}$, and the batch size set to 2.

CUHK-SYSU [26] is the first large dataset that can be used for a deep pedestrian retrieval system. The images are mainly from street surveillance and movie footage; one of the movie videos was selected from some of the pictures in the American comedy “Friends”. They contained a total of 18,184 images and 96,143 pedestrian frames. 8432 pedestrian frames were labeled with identities, and 87,711 pedestrian frames were not labeled with identities. Among them, 12,490 images were from surveillance, containing 6057 labeled identities; 5694 images were from movie footage, consisting of 2375 labeled identities. The whole dataset was divided into training and test sets, where the training set included 11,206 images and 5532 different identities; the test set consisted of 6978 images and 2900 query identities. The query pool of the test set had 6 different settings from 50 to 4000 people. The identities and images in both the training and test sets did not overlap.

The PRW [27] images were collected from 10 h of surveillance footage of Tsinghua University campus, captured by cameras from six different angles. A total of 11,816 images and 43,110 pedestrian frames were included, 34,304 pedestrian frames were marked as identity, and 8806 pedestrian frames were not marked as identity. The whole dataset was divided into two parts: the training set contained 5704 images and 482 different identities; the test set consisted of 6112 images and 2057 query images with 450 different identities. The PRW dataset used all the test set images as the query library, and the identities and images in the training and test sets did not overlap.

### 4.2. Measurement Standard

The evaluation index of pedestrian retrieval includes two components: the evaluation of detection box accuracy and the evaluation of re-recognition accuracy. There are two types: component heads and text heads. The cumulative matching feature (CMC) and average accuracy (mAP) were used as performance indexes. We used the same metrics (namely, average detection accuracy (mAP) and top-1 accuracy) to evaluate the performance of different methods in pedestrian retrieval. Average precision (AP) value (the percentage of positive samples that contains authentic samples is called precision) recall is the proportion of all real samples correctly predicted, as in:

(4) $$\mathrm{Precision}=\frac{TP}{TP+FP}\;$$

(5) $$\mathrm{Recall}=\frac{TP}{TP+FN}\;$$

### 4.3. Experimental Results

In this section, we examine the suggested method’s conclusive practical effectiveness on several datasets and evaluate it with the findings of other methodologies. To make the comparison more persuasive, the one-step and two-step procedures are examined separately.

Table 1 displays the outcomes of the various approaches on the CHUK-SYSU dataset. All the approaches in the table are illustrated with the ResNet-50 backbone. (i) The research methods were presented in diverse journals published in different years. The first essay appeared in 2018, whereas the most recent one appeared in 2022. (ii) In the context of mAP and Rank-1 accuracy, our experimental outcomes beat earlier techniques. The mAP was 96.23%, whereas the Rank-1 accuracy was 97.8%. The mAP was 1.43% superior to the baseline, whereas Rank-1 was 2.1% higher.

PRW may be a more difficult dataset than CHUK-SYSU. The factors contributing to this are (i) extreme perspective variety and impediment issues for the shorter tests in PRW and (ii) assurance of offsets within the bounding box explanations by target location procedures. All the approaches tabulated in Table 2 are as well located on the ResNet-50 backbone. The mAP value was 48.93% and the Rank-1 precision was 88.7%. The mAP value was 1.33% higher than the baseline; the Rank-1 value was 1.1% better than the baseline. It can be observed that both our mAP and Rank-1 were clearly superior to other approaches.

It can be observed from the outcomes of two experimental data sets and the comparison with other methods that the method proposed in this research is currently the most efficient.

### 4.4. Ablation Experiment

To ensure the accuracy of our method’s experiment results, we conducted an incremental evaluation of its modules on the CUHK-SYSU and PRW datasets. The experimental results when evaluating our model using these two datasets were more persuasive because they include complex scenarios and contain a lot of information. We again used ResNet-50 as the backbone network. In Figure 4, the feature fusion module (FFM) includes the output of three layers after the res2-res4 module output, and then fusion was carried out. We conducted experiments on each module in order to confirm in more detail the experimental effect of each module on the whole network. The results are shown in Table 3 and Table 4. For all three modules, removing any of the modules yielded lower results than combining them together. This indicates that the three modules are complementary.

After implementing the ICM on the CUHK-SYSU dataset, the mAP value improved by 0.8%, and after implementing the ICM on the PRW dataset, the mAP value improved by 0.4%, which shows that the module expands the acceptance domain of feature diagrams by inception convolution, gathers multi-scale contextual data, and extracts more useful pedestrian target features without sacrificing image data. The mAP value also increases when FFM is applied to the network, due to the capability of FFM to exploit high and low level characteristics in an appropriate way. It is suggested that these two modules work hand in hand in order to uncover more hidden features. The mAP value improved by 0.9% after instituting DHM in the CUHK-SYSU dataset and by 0.3% after performing DHM in the PRW dataset. This indicates that utilizing both branches in the double-headed structure helps to identify and gain better pedestrian recognition features. Therefore, our three suggested modules are justified by their effectiveness and reliability.

The GAN-CNN-TL algorithm [49] has the advantages of additional data generation, reduced biased detection model, automatic feature extraction, and enhanced hyperparameter tuning, which are common for ablation experiments. To more clearly demonstrate the effectiveness of our proposed method (IC-FFM), we introduced GAN-CNN-TL for further ablation experiments, and the experimental results on the CUHK-SYSU dataset and PRW dataset are shown in Table 5 and Table 6, respectively. We added GAN-CNN-TL to the backbone network and conducted ablation experiments with each of our proposed three modules, and the experimental results showed that our proposed method has complementary effects.

To illustrate the exploratory capabilities of our FFM, we evaluated an incremental assessment of its modules on the PRW dataset. The dataset incorporates various subtle elements and complex scenarios. In the expanded version, this dataset is captured by six diverse cameras and annotated with bounding boxes by the DPM finder. Here, ID loss and triplet loss were still present as the pattern for ResNet-50. As a result, when the model is subjected to testing, the effects are more persuading.

Within the plan of this paper, the include combination module performs distinctive highlight fusion on M2, M3, and M4. The feature outline yield within the initial layer of the ResNet-50 network model is huge, contains exceptionally complex data, and incorporates a huge number of parameters and a large amount of calculation. Thus, we performed highlight extraction and highlight combination after generating the highlight outline. For the purpose of confirming the effectiveness of the FFM, we performed the following tests on the PRW dataset. In these tests, the internal points of interest were changed and the results are shown in Table 7.

Table 7 shows the experimental outcomes of the feature fusion module’s fusion of different feature layer mappings. The output mAP was 47.2% after the feature fusion of M2 and M3, 47.6% after the feature fusion of M2 and M4, 47.9% after the fusion of M3 and M4, and 48.2% after the fusion of M2, M3, and M4. The experimental results show that the best performance was achieved after the fusion of M2, M3, and M4 outputs, which indicates that the experimental results mainly improved the complementary information from different modules and integrated it into a new feature. It is effective because the formation of new features has a certain positive effect on the advance of detailed information. Additionally, it provides the complementarity of information between different layers in order to produce more implicit features.

The above ablation experiments demonstrate the effect achieved by the number of features fused at different levels in the feature fusion module in this paper. In order to further verify the local and global effect, this paper set up another ablation experiment. The improved double-head method in the Faster R-CNN head module was tested. It has a full connector focusing on classification and a regression convolution header for the Bounding Box. It was added to the detection module and baseline re-ID module of the Faster R-CNN header module, respectively. Double head was added to both the detection and recognition phases before classification and regression. We conducted experiments on the PRW dataset, and the results are shown in Table 8.

Table 8 shows the experimental results after adding double-head classification and regression in different modules. The mAP was 47.9% when the Faster R-CNN header module was added alone, 47.2% when the re-ID module was added alone, and 47.5% when the Faster R-CNN header module and the re-ID module were combined. The results of the experiments demonstrate that the most effective experimental outcomes are only obtained by adding a double-head to the Faster R-CNN head module for classification and regression. In the later re-identification stage, more implicit features may be retrieved and higher accuracy can be obtained since the complementarity of information from different layers can be directly formed. It is therefore most efficient to incorporate the double-headed mechanism into the Faster R-CNN head module in this paper.

### 4.5. Visual Comparison

**Figure 5:**
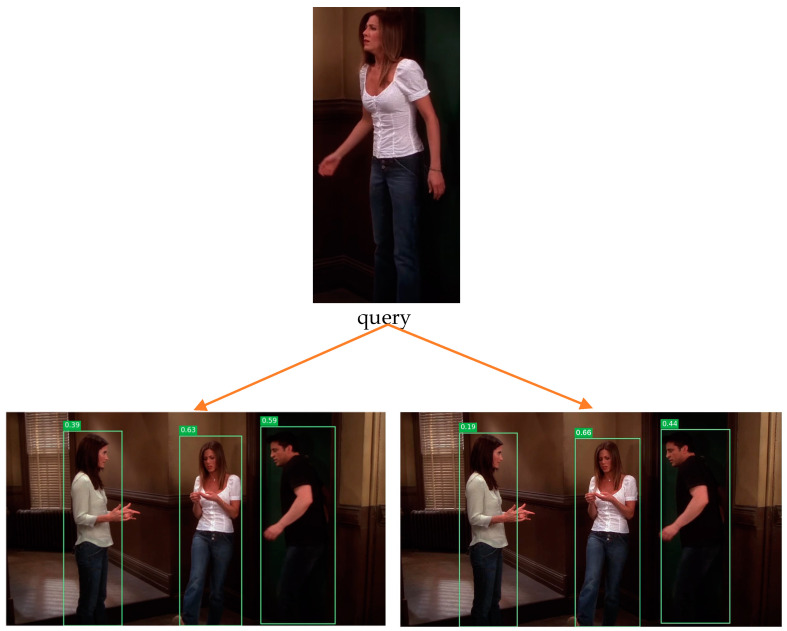

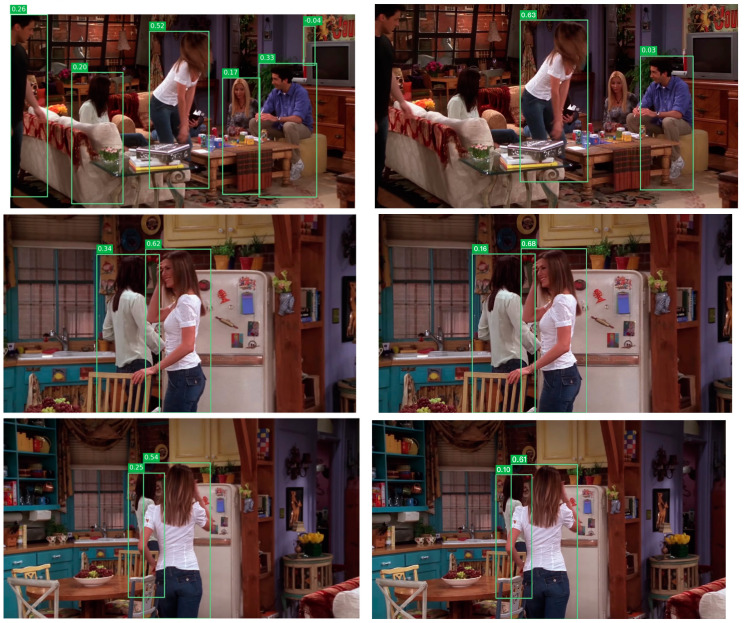

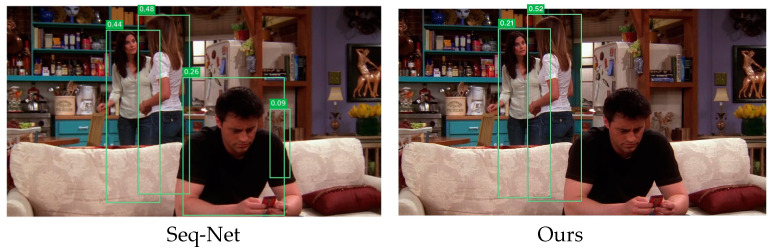
Visual comparison, the first row is the query image, the first column is the visualization results retrieved on the benchmark framework, and the second column is the visualization results retrieved by the proposed method.

The ablation experiment analysis in Section 4.5 demonstrates the reliability and efficacy of the model design used in this paper. In this section, in order to better understand the retrieval impact of the benchmark framework model and the model in this article, a visual comparison was undertaken. This paper randomly selected five images from the CHUK-SYSU dataset to demonstrate the retrieval effect. As shown in Figure 5, the first row is the pedestrian to be retrieved, the first column is the visualization of the baseline framework (Seq-Net) retrieval, and the second column is the visualization of the model framework (IC-FFM) retrieval proposed in this paper. By visualizing the results, the query image and the target viewpoint in the gallery were the same. Both our method and the Seq-Net test yielded correct results, but our method retrieved targets with higher accuracy than Seq-Net. However, the baseline framework (Seq-Net) results in errors when retrieving the incorrect target in a complicated scenario with many individuals and complex overlapping pedestrians. As shown in the second line, our method can effectively optimize the error detection rate with higher accuracy. In general, our model is more robust when the query target changes or crosses the camera. It can provide more discriminative features based on attributes and can more accurately retrieve the location of the target image in the query image. Our proposed model is more accurate than Seq-Net for person search.

## 5. Conclusions

For person search, we propose an inception convolution and feature fusion module (IC-FFM) algorithm to obtain more different features and information to improve detection accuracy. First, we proposed a method to expand the receptive field using the initial convolution module (ICM) instead of conventional convolution. This approach effectively increased the receptive field of features, forced the network to choose more pertinent pedestrian data globally, and increased the network’s capacity for recognition and retrieval accuracy. Then, we designed a local feature fusion module (FFM) to efficiently fuse the shallow information, such as location and shape at the bottom, and obtain efficient pedestrian information at various levels, improving the accuracy of recognition. Finally, we used double-head technology to develop a double-head module (DHM). This module can extract richer pedestrian features, learn pedestrian features better, improve the accuracy of re-identification, and it can also collect long-range aspects to make them more expressive. Extensive ablation researchers demonstrated our design’s superior efficiency and cutting-edge performance. Our model performs very well in dense pedestrian scenes, especially in terms of detail. However, our model is still limited by localization accuracy. I think the future research direction of person search is in the following areas: (1) a significant number of unlabeled or newly emerged pedestrians can be found in real scenes, and exploiting this information is an effective research direction; (2) the real-time performance of the person search network needs to be further improved so that it can be deployed in real-world scenarios; (3) although pedestrian retrieval integrates pedestrian detection and pedestrian re-identification tasks, how to make the two modules more efficient and how to make them better complement each other need to be further explored and studied. Therefore, in future work, the follow-up work of this paper is expected to further improve the results of pedestrian retrieval using a lightweight and effective anchorless mechanism on the pedestrian re-identification branch.

## Figures and Tables

**Table 1:** Comparison with the most cutting-edge methods on the CUHK-SYSU dataset (%).

Methods		mAP	Rank-1
one-step	OIM [33]	75.5	78.7
	IAN [34]	76.3	80.1
	NPSM [35]	77.9	81.2
	RCAA [36]	79.3	81.3
	CTXG [37]	84.1	86.5
	QEEPS [38]	88.9	89.1
	HOIM [39]	89.7	90.8
	BINet [40]	90.0	90.7
	NAE [30]	91.5	92.4
	NAE+ [30]	92.1	92.9
	PGA [41]	92.3	94.7
	DKD [42]	93.1	94.2
	LMDP [49]	92.9	94.3
	AlignPS [17]	93.1	94.2
	AlignPS+ [17]	94.0	94.5
	AGWF [43]	93.3	94.2
	Baseline	94.8	95.7
	**ours**	**96.2**	**97.8**
two-step	DPM [44]	-	-
	MGTS [45]	83.0	83.7
	CLSA [46]	87.2	88.5
	RDLR [8]	93.0	94.2
	IGPN [11]	90.3	91.4
	OR [39]	92.3	93.8
	TCTS [26]	93.9	95.1

**Table 2:** Comparison with the most cutting-edge methods on the PRW dataset (%).

Methods		mAP	Rank-1
one-step	OIM [33]	21.3	49.4
	IAN [34]	23.0	61.9
	NPSM [35]	24.2	53.1
	RCAA [36]	-	-
	CTXG [37]	33.4	73.6
	QEEPS [38]	37.1	76.7
	HOIM [39]	39.8	80.4
	BINet [40]	45.3	81.7
	NAE [30]	43.3	80.9
	NAE+ [30]	44.0	81.1
	PGA [41]	44.2	85.2
	DKD [42]	50.5	87.1
	LMDP [49]	52.3	87.9
	AlignPS [17]	45.9	81.9
	AlignPS+ [17]	46.1	82.1
	AGWF [43]	53.3	87.7
	Baseline	47.6	87.6
	**ours**	**48.9**	**89.7**
two-step	DPM [44]	20.5	48.3
	MGTS [45]	32.6	72.1
	CLSA [46]	38.7	65.0
	RDLR [8]	42.9	70.2
	IGPN [11]	47.2	87.0
	OR [39]	52.3	71.5
	TCTS [26]	46.8	87.5

**Table 3:** Impact of each module on network performance on the CUHK-SYSU dataset (%).

ICM	FFM	DHM	mAP	Rank-1	Rank-5	Rank-10
-	-	-	94.8	95.7	-	-
√	-	-	95.6	96.4	95.9	97.4
-	√	-	95.8	97.1	96.3	97.9
-	-	√	95.7	96.9	96.1	97.7
√	√	-	96.0	97.4	97.1	98.4
√	-	√	95.9	97.2	96.9	98.2
-	√	√	96.1	97.5	98.1	98.5
√	√	√	**96.2**	**97.8**	**98.5**	**98.9**

**Table 4:** Impact of each module on network performance on the PRW dataset (%).

ICM	FFM	DHM	mAP	Rank-1	Rank-5	Rank-10
-	-	-	47.6	87.6	-	-
√	-	-	48.0	88.1	95.8	96.3
-	√	-	48.2	88.3	96.0	96.8
-	-	√	47.9	87.9	95.6	96.1
√	√	-	48.2	88.9	96.2	96.5
√	-	√	48.1	88.6	96.1	96.3
-	√	√	48.5	89.2	96.3	96.9
√	√	√	**48.9**	**89.7**	**96.5**	**97.3**

**Table 5:** Impact of GAN-CNN-TL and modules on the network performance of the CUHK-SYSU dataset after adding (%).

GAN-CNN-TL	ICM	FFM	DHM	mAP	Rank-1	Rank-5	Rank-10
-	-	-	-	94.8	95.7	-	-
	√	-	-	95.6	96.4	95.9	97.4
	-	√	-	95.8	97.1	96.3	97.9
	-	-	√	95.7	96.9	96.1	97.7
√	-	-	-	94.6	96.5	95.8	97.5
	√	√	-	96.0	97.4	97.1	98.4
	√	-	√	95.9	97.2	96.9	98.2
	-	√	√	96.1	97.5	98.1	98.5
√	√	-	-	94.3	94.4	94.9	96.7
√	-	√	-	94.6	94.9	95.3	96.9
√	-	-	√	94.9	95.1	95.5	97.1
√	√	√	-	95.2	96.6	95.9	97.3
√	√	-	√	95.0	96.2	96.3	97.6
√	-	√	√	95.6	96.9	97.0	97.8
√	√	√	√	95.8	97.2	97.8	98.1
-	√	√	√	**96.2**	**97.8**	**98.5**	**98.9**

**Table 6:** Impact of GAN-CNN-TL and modules on the network performance of the PRW dataset after adding (%).

GAN-CNN-TL	ICM	FFM	DHM	mAP	Rank-1	Rank-5	Rank-10
-	-	-	-	47.6	87.6	-	-
	√	-	-	48.0	88.1	95.8	96.3
	-	√	-	48.2	88.3	96.0	96.8
	-	-	√	47.9	87.9	95.6	96.1
√	-	-	-	45.6	84.4	92.8	93.3
	√	√	-	48.2	88.9	96.2	96.5
	√	-	√	48.1	88.6	96.1	96.3
	-	√	√	48.5	89.2	96.3	96.9
√	√			45.8	84.9	93.1	93.6
√		√		46.0	84.7	93.3	96.8
√			√	45.7	84.2	92.5	93.1
√	√	√		46.3	85.4	93.8	94.0
√	√		√	46.6	85.9	94.0	94.6
√		√	√	47.1	86.3	95.4	95.7
√	√	√	√	47.6	88.1	95.8	95.9
-	√	√	√	**48.9**	**89.7**	**96.5**	**97.3**

**Table 7:** Impact of different module fusion on network performance on the PRW dataset (%).

M2	M3	M4	mAP	Rank-1	Rank-5	Rank-10
		√	46.3	86.1	93.2	93.9
	√		46.6	86.4	93.8	94.3
√			46.9	86.8	94.3	95.1
√	√	-	47.2	87.1	94.9	95.6
√	-	√	47.6	87.7	95.2	95.9
-	√	√	47.9	87.9	95.7	96.4
√	√	√	**48.2**	**88.3**	**96.0**	**96.8**

**Table 8:** Impact of adding double-head module (DHM) to different modules on network performance on the PRW dataset (%).

Faster R-CNN	re-ID	mAP	Rank-1	Rank-5	Rank-10
√	-	**47.9**	**87.9**	**95.6**	**96.1**
-	√	47.2	87.1	95.1	95.6
√	√	47.5	87.6	95.3	95.8

## Data Availability

Not applicable.

## References

[1] Girshick R., Donahue J., Darrell T., Malik J. Rich feature hierarchies for accurate object detection and semantic segmentation. Proceedings of the IEEE Conference on Computer Vision and Pattern Recognition.

[2] Girshick R., Iandola F., Darrell T., Malik J. Deformable part models are convolutional neural networks. Proceedings of the IEEE Conference on Computer Vision and Pattern Recognition.

[3] Yang Y., Wen L., Lyu S., Li S.Z. Unsupervised learning of multi-level descriptors for person reidentification. In Proceedings of the Thirty-First AAAI Conference on Artificial Intelligence.

[4] Zhao C., Wang X., Chen Y., Gao C., Zuo W., Miao D. Consistent iterative multi-view transfer learning for person re-identification. Proceedings of the IEEE International Conference on Computer Vision Workshops.

[5] Wang G., Lai J., Huang P., Xie X. Spatialtemporal person re-identification. Proceedings of the AAAI Conference on Artificial Intelligence.

[6] Fu Y., Wei Y., Zhou Y., Shi H., Huang G., Wang X., Yao Z., Huang T. Horizontal pyramid matching for person re-identification. Proceedings of the AAAI Conference on Artificial Intelligence.

[7] Hao Y., Wang. N., Li J., Gao X. (2019). HSME: Hypersphere manifold embedding for visible thermal person reidentification. Proceedings of the AAAI Conference on Artificial Intelligence.

[8] Zhao C., Lv X., Zhang Z., Zuo W., Wu J., Miao D. (2020). Deep fusion feature representation learning with hard mining center-triplet loss for person re-identification. IEEE Trans. Multi-Media.

[9] Dong W., Zhang Z., Song C., Tan T. Instance guided proposal network for person search. Proceedings of the 2020 IEEE/CVF Conference on Computer Vision and Pattern Recognition.

[10] Lan X., Zhu X., Gong S. Person search by multi-scale matching. Proceedings of the European Conferenceon Computer Vision (ECCV).

[11] Munjal B., Amin S., Tombari F., Galasso F. Query-guided end-to-end person search. Proceedings of the IEEE Conference on Computer Vision and Pattern Recognition (CVPR).

[12] Wang C., Ma B., Chang H., Shan S., Chen X. TCTS: A task-consistent two-stage framework for person search. Proceedings of the IEEE Conference on Computer Vision and Pattern Recognition (CVPR).

[13] Xu Y., Ma B., Huang R., Lin L. Person search in a scene by jointly modeling people commonness and person uniqueness. Proceedings of the 22nd ACM International Conference on Multimedia, MM ’14.

[14] Chen Z., Huang S., Tao D. Context refinement for object detection. Proceedings of the European Conference on Computer Vision (ECCV).

[15] Liu J., Zha Z.J., Hong R., Wang M., Zhang Y. Dual Context-Aware Refinement Network for Person Search. Proceedings of the 28th ACM International Conference on Multimedia, MM ’20.

[16] Xiao T., Li S., Wang B., Lin L., Wang X. Joint detection and identification feature learning for person search. Proceedings of the IEEE Conference on Computer Vision and Pattern Recognition (CVPR).

[17] Yan Y., Li J., Qin J., Bai S., Liao S., Liu L., Zhu F., Shao L. Anchor-free person search. Proceedings of the IEEE Conference on Computer Vision and Pattern Recognition (CVPR).

[18] Yang W., Li D., Chen X., Huang K. Bottom-Up Foreground-Aware Feature Fusion for Person Search. Proceedings of the 28th ACM International Conference on Multimedia, MM ’20.

[19] Fan B., Wang L., Zhang R., Guo Z., Zhao Y., Li R., Gong W. Contextual Multi-Scale Feature Learning for Person Re-Identification. Proceedings of the 28th ACM International Conference on Multimedia, MM ’20.

[20] Huang Y., Zha Z.J., Fu X., Zhang W. Illumination-invariant person re-identification. Proceedings of the 27th ACM International Conference on Multimedia, MM ’19.

[21] Wang G., Yuan Y., Chen X., Li J., Zhou X. Learning discriminative features with multiple granularities for person re-identification. Proceedings of the 26th ACM International Conference on Multimedia, MM ’18.

[22] Xiao J., Xie Y., Tillo T., Huang K., Wei Y., Feng J. (2019). IAN: The individual aggregation network for person search. Pattern Recognit..

[23] Ren S., He K., Girshick R., Sun J. Faster R-CNN: Towards real-time object detection with region proposal networks. Proceedings of the Advances in Neural Information Processing Systems 28 (NIPS 2015).

[24] Han C., Ye J., Zhong Y., Tan X., Zhang C., Gao C., Sang N. Re-id driven localization refinement for person search. Proceedings of the IEEE Conference on Computer Vision and Pattern Recognition (CVPR).

[25] Chen D., Zhang S., Yang J., Schiele B. Norm-Aware Embedding for Efficient Person Search. Proceedings of the IEEE Conference on Computer Vision and Pattern Recognition (CVPR).

[26] Zheng L., Zhang H., Sun S., Chandraker M., Yang Y., Tian Q. Person re-identification in the wild. Proceedings of the IEEE Conference on Computer Vision and Pattern Recognition (CVPR).

[27] Liu H., Feng J., Jie Z., Jayashree K., Zhao B., Qi M., Jiang J., Yan S. Neural Person Search Machine. Proceedings of the IEEE Conference on Computer Vision and Pattern Recognition (CVPR).

[28] Chang X., Huang P.Y., Shen Y.D., Liang X., Yang Y., Hauptmann A.G. RCAA: Relational context-aware agents for person search. Proceedings of the European Conference on Computer Vision (ECCV).

[29] Yan Y., Zhang Q., Ni B., Zhang W., Xu M., Yang X. Learning context graph for person search. Proceedings of the IEEE Conference on Computer Vision and Pattern Recognition (CVPR).

[30] Chen D., Zhang S., Ouyang W., Yang J., Schiele B. Hierarchical online instance matching for person search. Proceedings of the AAAI Conference on Artificial Intelligence.

[31] Dong W., Zhang Z., Song C., Tan T. Bi-directional interaction network for person search. Proceedings of the IEEE Conference on Computer Vision and Pattern Recognition (CVPR).

[32] Kim H., Joung S., Kim I.J., Sohn K. Prototype-guided saliency feature learning for person search. Proceedings of the IEEE Conference on Computer Vision and Pattern Recognition (CVPR).

[33] Zhang X., Wang X., Bian J.W., Shen C., You M. Diverse knowledge distillation for end-to-end person search. Proceedings of the AAAI Conference on Artificial Intelligence.

[34] Han B.J., Ko K., Sim J.Y. End-to-end trainable trident person search network using adaptive gradient propagation. Proceedings of the IEEE Conference on Computer Vision and Pattern Recognition (CVPR).

[35] Chen D., Zhang S., Ouyang W., Yang J., Tai Y. (2020). Person search by separated modeling and A maskguided two-stream CNN model. IEEE Trans. Image Processing.

[36] Li Z., Miao D. Sequential end-to-end network for efficient person search. Proceedings of the AAAI Conference on Artificial Intelligence.

[37] Zhang Y., Chu J., Leng L., Miao J. (2020). Mask Refined R-CNN: A network for refining object details in instance segmentation. Sensors.

[38] Chu J., Guo Z., Leng L. (2018). Object detection based on multi-layer convolution feature fusion and online hard example mining. IEEE Access.

[39] Leng L., Li M., Kim C., Bi X. (2017). Dual-source discrimination power analysis for multi-instance contactless palmprint recognition. Multimed. Tools Appl..

[40] Zhang Y., Wang S., Kan S., Cen Y., Zhang L. (2023). End-to-end feature diversity person search with rank constraint of cross-class matrix. Neurocomputing.

[41] Gu H., Li J., Fu G., Yue M., Zhu J. (2022). Loss function search for person re-identification. Pattern Recognit..

[42] Lv N., Xiang X., Wang X., Yang J., Abdein R. (2022). Efficient person search via learning-to-normalize deep representation. Neurocomputing.

[43] Valem P.L., Pedronette G.D.C. (2022). Person Re-ID through unsupervised hypergraph rank selection and fusion. Image Vis. Comput..

[44] Li Y., Chen Y. (2022). Infrared-visible cross-modal person re-identification via dual-attention collaborative learning. Signal Processing: Image Commun..

[45] Yang M., Liao L., Ke K., Gao G. (2022). Multi-feature sparse similar representation for person identification. Pattern Recognit..

[46] Naushad R., Kaur T., Ghaderpour E. (2021). Deep Transfer Learning for Land Use and Land Cover Classification: A Comparative Study. Sensors.

[47] Chen J.C., Wu C.F., Chen C.H., Lin C.R. (2020). Person Search via Deep Integrated Networks. Appl. Sci..

[48] Fiaz M., Cholakkal H., Narayan S., Anwer R.M., Khan F.S. PS-ARM: An End-to-End Attention-aware Relation Mixer Network for Person Search. Proceedings of the IEEE Conference on Computer Vision and Pattern Recognition (CVPR).

[49] Chui K.T., Gupta B.B., Alhalabi W., Alzahrani F.S. (2022). An MRI Scans-Based Alzheimer’s Disease Detection via Convolutional Neural Network and Transfer Learning. Diagnostics.

